# Suppression of VAMP2 Alters Morphology of the Tegument and Affects Glucose uptake, Development and Reproduction of *Schistosoma japonicum*

**DOI:** 10.1038/s41598-017-05602-8

**Published:** 2017-07-12

**Authors:** Qian Han, Bingguang Jia, Yang Hong, Xiaodan Cao, Qi Zhai, Ke Lu, Hao Li, Chuangang Zhu, Zhiqiang Fu, Yonghong Shi, Jiaojiao Lin

**Affiliations:** 1 Ministry of Agriculture, Shanghai Veterinary Research Institute, Chinese Academy of Agricultural Sciences, Shanghai, People’s Republic of China; 2 Jiangsu Co-innovation Center for Prevention and Control of Important Animal Infectious Diseases and Zoonoses, Yangzhou, China

## Abstract

Schistosomiasis caused by schsitosomes is a serious global public health concern. The tegument that surrounds the worm is critical to the schistosomes survival. The tegument apical membrane undergoes a continuous process of rupture and repair owing to membranous vacuoles fusing with the plasma membrane. Vesicle-associated membrane protein 2 (VAMP2), a member of soluble N-ethylmaleimide sensitive factor attachment protein receptor (SNAREs) is required for membrane fusion. Here, we used RNA interference (RNAi) to knock down the expression of VAMP2 of *Schistosoma japonicum* (*SjVAMP2*), and both real-time PCR and western blot analysis confirmed the suppression of this molecule, as well as the suppression of the transcript levels of schistosome glucose transporters (*SGTP1* and *SGTP4*), and insulin receptors (*SjIR1* and *SjIR2*). *SjVAMP2*-suppressed worms exhibited a lower viability, and phenotypic alterations were also observed in the tegument. Moreover, the glucose consumption of *SjVAMP2*-suppressed worms decreased significantly in 4 and 6 days, respectively, as well as a significant reduction in egg production. We also observed a significant reduction in worm burden and hepatic eggs burden in two independent RNAi experiment *in vivo*, and minor pathological changes in mice treated with *SjVAMP2* specific small interfering (si)RNA. These findings reveal that *SjVAMP2* may play important roles in the maintenance of tegument, glucose uptake, worm development and egg production in schistosomes.

## Introduction

Schistosomiasis is a tropical parasitic disease that infects approximately 200 million people, and approximately 700 million people are at risk in 74 countries; it is caused by blood-dwelling fluke worms of the genus *Schistosoma*. Schistosomes have a complex life cycle that involves snails as intermediate hosts, and mammals (including humans) as definitive hosts^1, 2^. After cercariae penetrate the skin, they transform into young worms known as schistosomula, move into the bloodstream, and mature. After sexual maturation, adult schistosomes mate and lay eggs, and the eggs are primarily responsible for the pathological damage and disease transmission^3, 4^. Paired male and female schistosomes reside in the mesenteric veins of mammalian hosts and can survive for decades surrounded by the components of the immune system. Therefore, these organisms must have sophisticated mechanisms to evade host immune effectors and use host nutrients for metabolic processes and growth^5^.

The entire worm is surrounded by a continuous cytoplasmic membrane, or syncytium, known as the tegument which is the direct interface between the parasite and its host^6^. The tegument is believed to be pivotal in evading hostile immune responses via antigenic mimicry^7, 8^, proteolytic degradation of “attacking” host proteins^9^, rigid biophysical membrane properties^10^ and a rapid tegumental membrane turnover^11, 12^. Besides, the tegument is an important site of nutrient uptake from the host, such as glucose^13, 14^, amino acids and cholesterol^15, 16^, and also an ideal target site for the drugs^17^. Beneath the surface membrane, the tegumental cytoplasmic layer contains several kinds of secretory vesicles, including membranous bodies and discoid bodies, which contribute to the metabolism of worm and plasma membrane renew. The cell bodies of the tegument contain the machinery for protein synthesis, including the syncytium, endoplasmic reticulum, and Golgi apparatus, and produce several vesicular products, which are trafficked to the tegument along cytoplasmic bridges^18^ (Supplementary Figure S1). It is of note that the tegumental surface is an unusual heptalaminate membrane, consisting of two closely apposed lipid bilayers in the form of a normal plasma membrane, and is continually undergoing rupture and repair^19^. Studies have shown that membranous bodies might help form the outer bilayer when their bounding membrane is fused with the plasma membrane of the tegument in a conventional process of exocytosis^11, 20^.

Fusion of biological membranes is fundamental for the transport of cargo molecules, such as trafficked proteins, hormones, and neurotransmitters via vesicles trafficking in cell growth, membrane repair, cytokinesis and synaptic transmission process. The core proteins implicated in this process are soluble N-ethylmaleimide sensitive factor attachment protein receptor (SNARE) localized in opposing membranes, including synaptobrevin/Vesicle-associated membrane protein 2 (VAMP2) on the vesicle membrane (v-SNARE), syntaxin and 25-kDa synaptosome-associated protein (SNAP-25) families on the target membrane (t-SNARE). The formation of four-helix bundle, one from v-SNARE and three from t-SNARE, drives membrane fusion. VAMP2 (v-SNARE) has been extensively studied in neurons and confirmed to play a critical role in the fast exocytosis of neurotransmitter^21–23^. In non-neuronal tissues, VAMP2 is involved in the regulation of exocytosis including insulin secretion in pancreatic β-cells^24^, fusion of lytic granules in cytotoxic T-lymphocytes^25^, insulin-dependent translocation of GLUT4-containing vesicles in adipocytes^26, 27^ and muscle regeneration in quiescent satellite cells^28, 29^.

Schistosomes need large quantities of glucose for growth and reproduction, particularly during mating, and a considerable amount of energy for egg production^30^. Schistosomes uptake glucose directly from the host through the tegument and not through the gut^31^. Glucose is the common energy source for cellular metabolism and all cells absorb glucose through hydrophobic surface membranes using glucose transporter proteins (GTPs). In schistosomes, glucose is uptaken via two GTPs: *S*GTP1 and *S*GTP4^32–34^, and it has been reported that the suppression of genes *SGTP1* or *SGTP4* in *Schistosoma mansoni* (*S*. *mansoni*) using RNAi impairs the ability to intake glucose^35^. Glucose transporter type 4 (GLUT4) is the insulin-regulated glucose transporter primarily found in adipose tissues and striated muscle^36^. In response to insulin, GLUT4-containing vesicles colocalized with VAMP2 fuse with the plasma membrane of insulin-sensitive cells to stimulate glucose uptake. Numerous studies have revealed the importance of VAMP2 in GLUT4 translocation^24, 37, 38^. Study has shown that VAMP2 is located in an insulin-sensitive GLUT4 compartment and that the integrity of VAMP2 is necessary for the incorporation of GLUT4 vesicles into the cell surface in response to insulin^39^. Knockdown of VAMP2 affected insulin-induced GLUT4 translocation and glucose uptake, suggesting that VAMP2 is an important mediator of these processes^40^.

Genomic and proteomic analyses have allowed the identification of the *VAMP2* gene in *S*. *japonicum* (hereafter *SjVAMP2*)^41, 42^. Our research group cloned this gene and showed that *SjVAMP2* was primarily expressed in the tegument of schistosomes; in addition, its transcripts were highly expressed in 42-day female worms^43^. Despite these promising results, the biological functions of *SjVAMP2* have not been fully elucidated in *S*. *japonicum*. In this study, we employed the RNA interference (RNAi) technique^44^ to explore the functional role of *SjVAMP2* in worm growth, development, and female fecundity.

## Results

### Immunolocalization of SjVAMP2 on the tegument of schistosomes

Gold particles immunoreactive for SjVAMP2 were observed on the surface membrane of the tegument, particularly in its invaginations (Supplementary Figure S2A). The basal membrane and the underlying muscles were also immunostained. In the tegumental matrix, gold particles were specifically located on secretory vesicles, discoid bodies and membranous bodies in *S*. *japonicu*m (Supplementary Figure S2B).

### RNAi-induced knockdown of *SjVAMP2* gene expression *in vitro*

**Figure 1:**
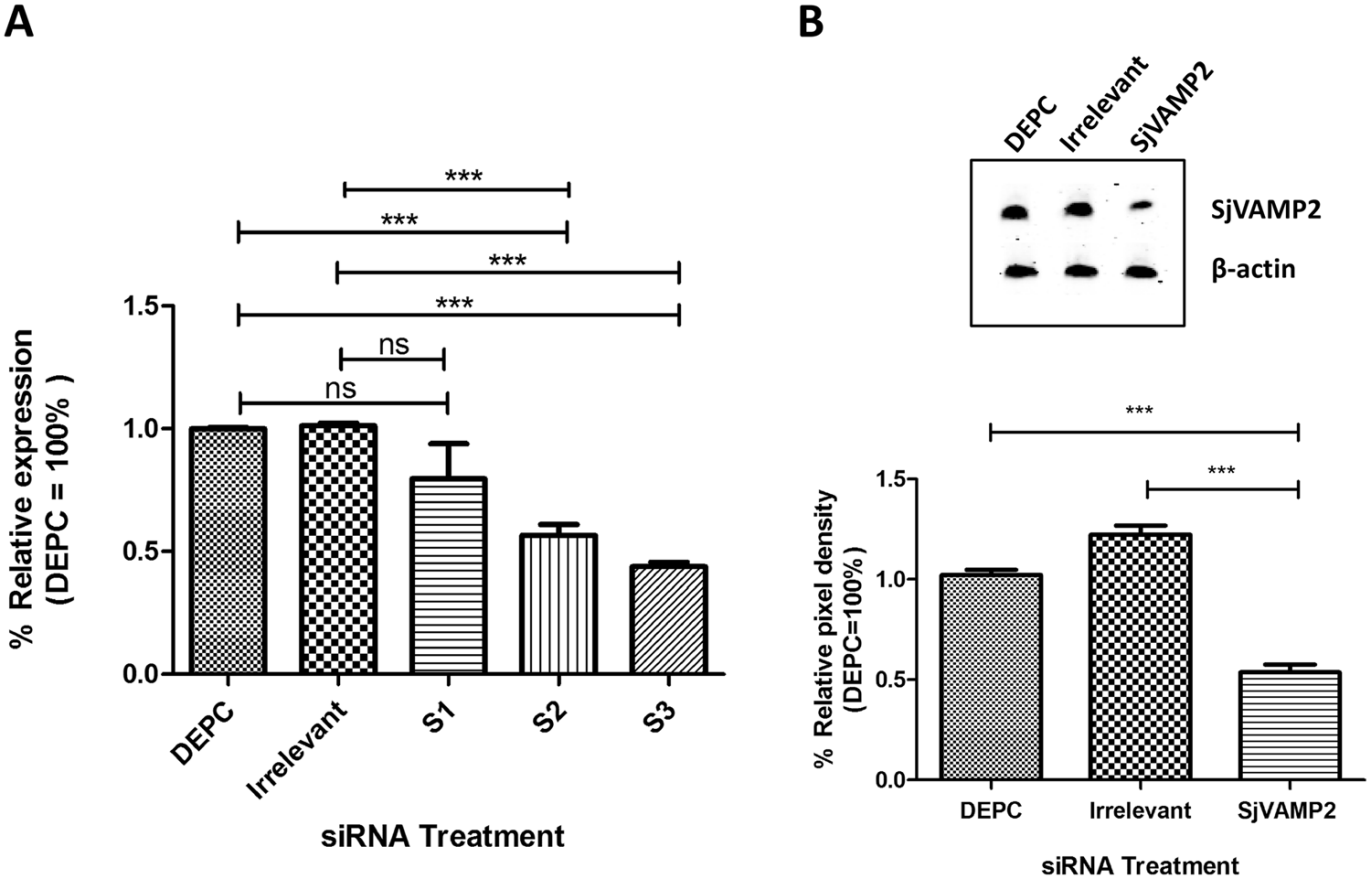
*SjVAMP2* gene expression analysis. (**A**) Relative gene expression of *SjVAMP2* in parasites 6 days after treatment with DEPC ultra pure water, irrelevant siRNA or three *SjVAMP2* specific siRNAs (S1, S2, and S3). (**B**) Protein levels in worm extracts obtained 6 days following treatment with the indicated S3 siRNA. Western blotting results of *Sj*VAMP2 protein were shown in the top panel with β-actin protein as loading control. Ratio of the optical density of *Sj*VAMP2 to β-actin was analyzed by imageJ^89^. Data show the mean and standard errors derived from three independent experiments (***P < 0.001).

Three different siRNAs (S1, S2, S3) sequences that targeted distinct regions of the *SjVAMP2* gene were synthesized (Supplementary Table S1), as well as the primers for real-time PCR analysis (Supplementary Table S2). Parasites obtained from mice 21 day post infection (dpi) were cultured in complete RPMI 1640 medium with *SjVAMP2*-specific siRNAs, irrelevant siRNA or diethyl pyrocarbonate (DEPC) water, respectively. After 6 days, worms in the three groups were harvested for the subsequent analysis. S1, S2, and S3 siRNA all silenced *SjVAMP2*, with 21.5%, 44.0% (P < 0.001), and 56.3% (P < 0.001) compared to the DEPC group, respectively. There was no significant difference in gene silencing between the irrelevant siRNA and DEPC group (Fig. 1A).

To confirm the impact of gene suppression on target protein levels, western blotting analysis was undertaken. Our results showed that the expression of SjVAMP2 protein significantly decreased in S3 siRNA treatment group compared with the control groups (P < 0.01), and the expression levels of β-actin (protein loading control) showed no significant difference between the three groups (Fig. 1B). Considering that S3 siRNA suppressed the expression of *SjVAMP2* at the transcriptional and translational levels significantly, this siRNA was selected for use in all subsequent experiments.

**Figure 2:**
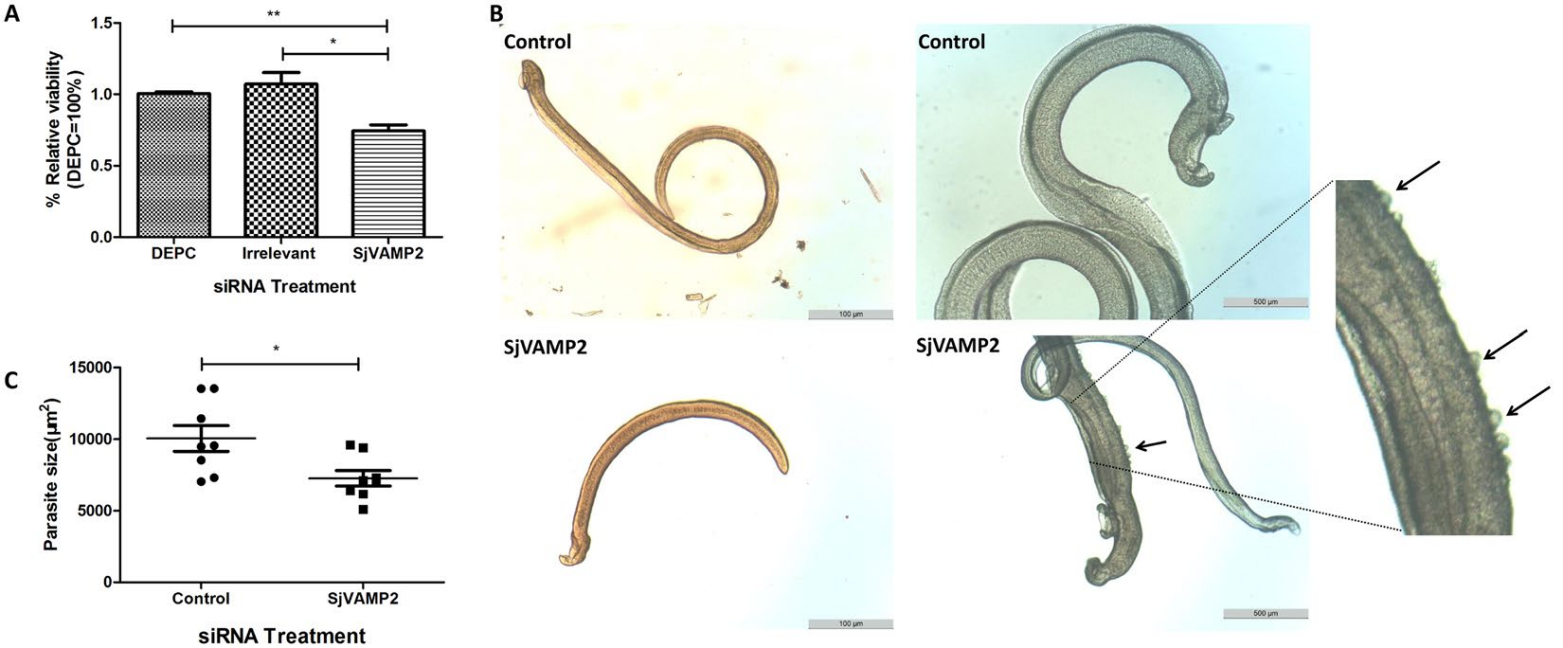
Relative viability and morphological changes of schistosomes treated with *SjVAMP2* specific siRNA. (**A**) The viability of worms was compared to DEPC control group from three independent experiments. (**B**) The morphological changes of worms were captured via optical microscopy. Images were derived from 3 randomly selected male worms from each group. Arrows indicated bulb-like structures on the surface of some male schistosomes. (**C**) The size of male schistosomes was analyzed by imageJ. Data was compared with DEPC control (*P < 0.05, **P < 0.01).

The effect of *SjVAMP2* suppression on parasite development was evaluated by assessing parasites viability and morphological changes at day 6 after siRNA treatment. Our results indicated that viability of *SjVAMP2*-suppressed parasites *in vitro* was 69.7% compared with that of the DEPC control group (P < 0.01) (Fig. 2A). In the worms treated with *SjVAMP2* siRNA, about 62% male worms exhibited significant morphological changes, mostly characterized by a smaller size (P < 0.01) (Fig. 2B,C), and some presented bubble-like structures on the surface of the anterior region (Fig. 2B). Morphological changes in the female worms were not observed.

### Effect of *SjVAMP2* suppression on the tegument

**Figure 3:**
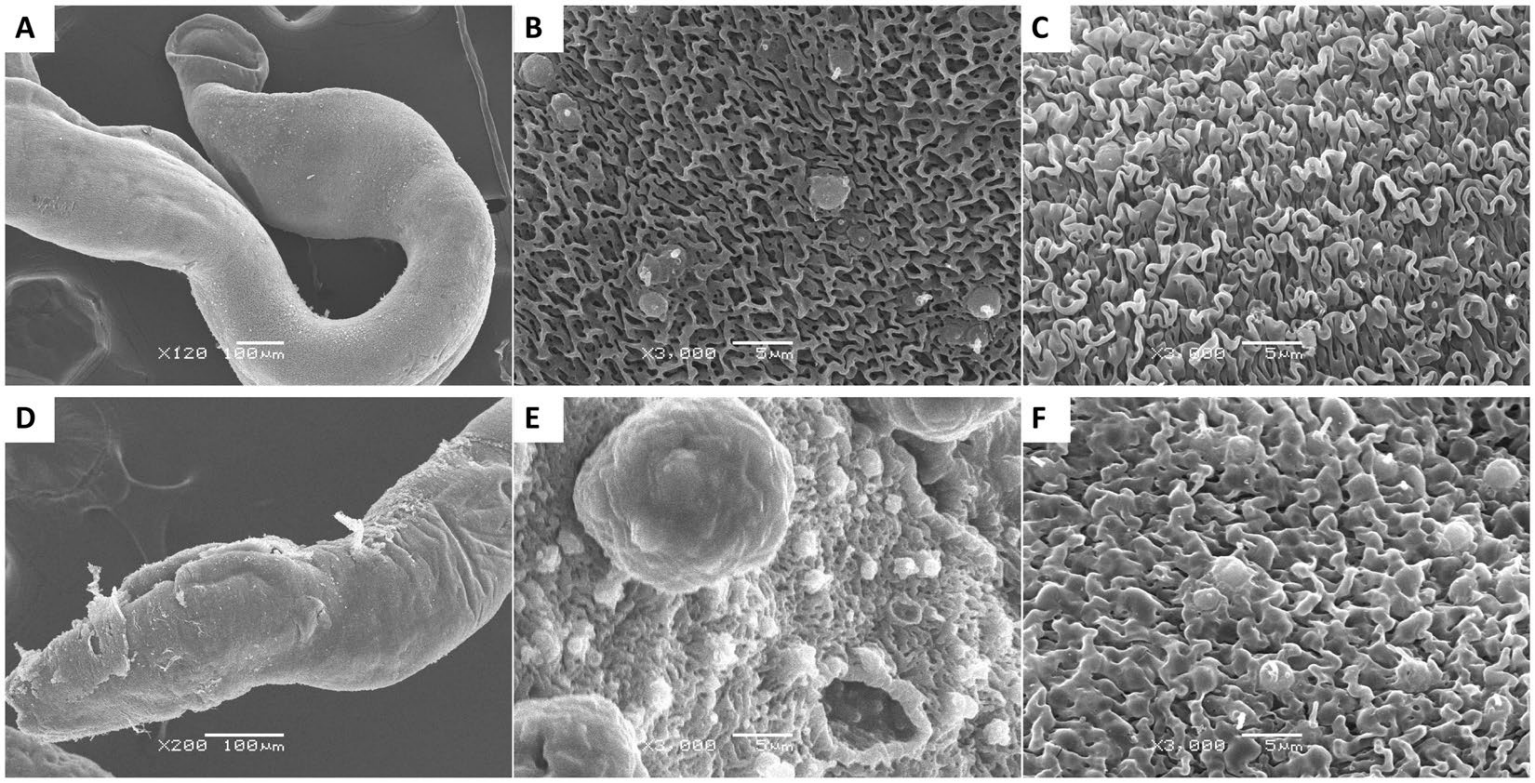
Scanning electron microscopy analysis of schistosomes after siRNA treatment. (**A**,**B**,**C**) Morphology of *S*. *japonicum* in the control group. (**A**) Anterior portion of a male schistosome under low magnification (120×). (**B**) Anterior portion of a male schistosome indicating the presence of a few crests and sensory papillae (3000×). (**C**) Highly-folded medial and posterior portions of a male schistosome showing a sponge-like appearance (3000×). (**D**,**E**,**F**) Morphology of male schistosomes treated with SjVAMP2 siRNA. (**D**) Peeling of the anterior region of the tegument of a male schistosome (200×). (**E**) Tegument with many abnormally oversized bulb-like structures, some of which collapsed (3000×). (**F**) Medial and posterior portions of the tegument with fused sponge-like ridges (3000×).

To further explore the phenotypic changes induced by *SjVAMP2* suppression, the morphology of the tegument in *SjVAMP2* siRNA-treated and control worms was evaluated by scanning electron microscopy (SEM) and transmission electron microscopy (TEM). Significant alterations in the tegumental and subtegumental tissues were observed in 67% *SjVAMP2*-suppressed worms. The morphology of the tegumental surface of adult worms in the DEPC control group was similar to that described previously^18, 45^. The integrity of the tegument was preserved (Fig. 3A), and low ridges and channels with sensory papillae was observed in the anterior region of male schistosomes (Fig. 3B) whereas the surface of the medial and posterior regions was highly folded, resulting in a sponge-like appearance (Fig. 3C). In *SjVAMP2*-suppressed male worms, tegument peeling occurred on the surface of the anterior portion (Fig. 3D). Oversized bulbs were also observed, some of which collapsed, leading to the formation of hole-like structures (Fig. 3E). In addition, the ridges fused together to form a mass in the dorsal medial region of male worms (Fig. 3F). In female worms, no substantial difference in the morphology of the tegumental surface was observed between the SjVAMP2 siRNA-treated and control groups.

**Figure 4:**
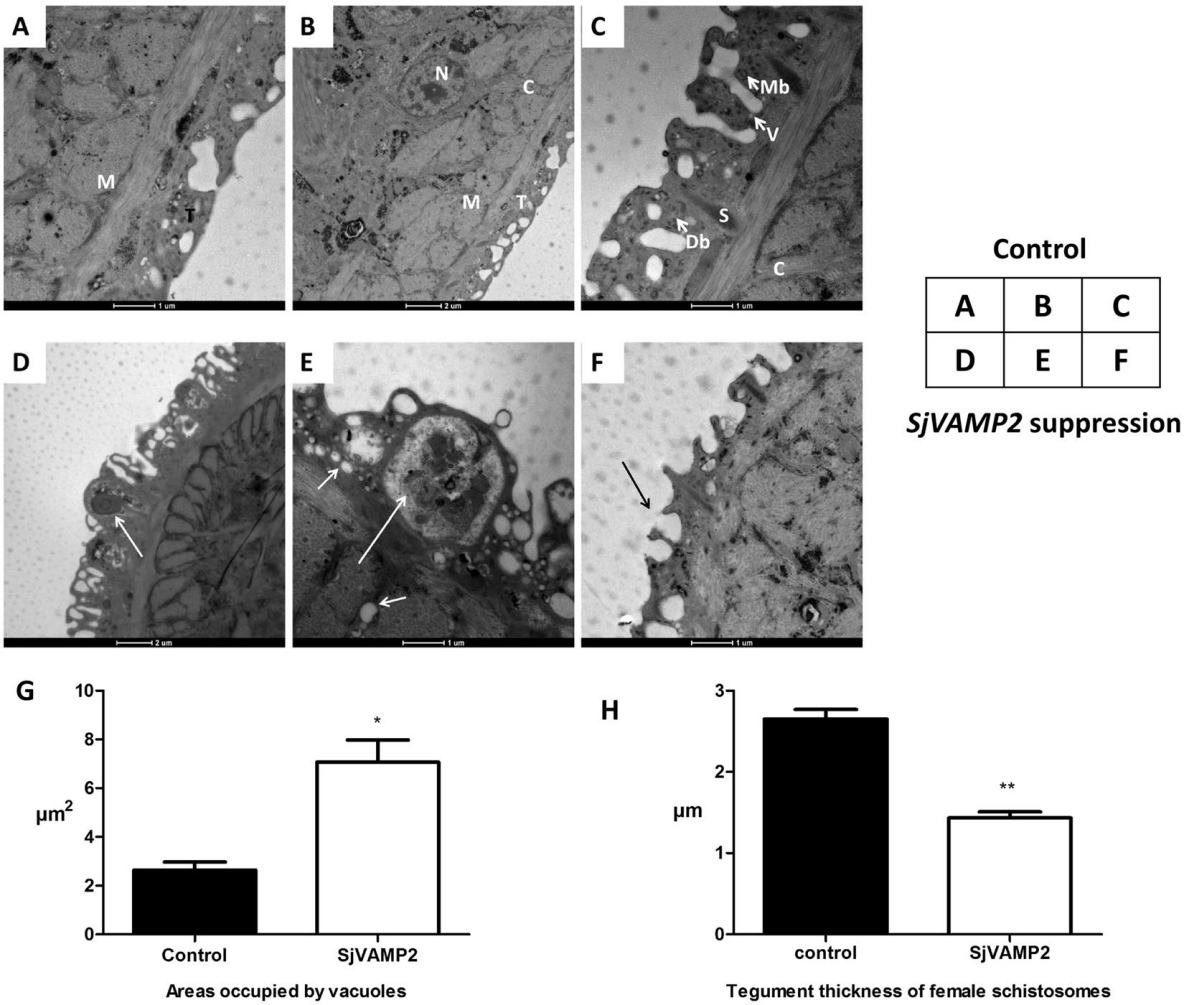
Transmission electron microscopy analysis of schistosomes after siRNA treatment. (**A**,**B**,**C**) Topography of *S*. *japonicum* in the control group, showing the whole tegument from the surface to the muscle layer. (**A**) The dorsal aspect of the female schistosome. (**B**,**C**) The dorsal aspect of the male schistosome. Tegument matrix (T), cytoplasmic bridge (C), nucleus of syncytium (N), spines (S), muscles (M) beneath the tegument, discoid bodies (Db), membranous bodies (Mb),vesicles (V) in the tegument matrix. (**D**,**E**,**F**) Topography of the tegument of *S*. *japonicum* after treatment with *SjVAMP2* specific siRNA. (**D**) Emergence of enlarged membranous bodies with hepatalmemebrane in the tegument matrix of male worm (arrow indicated). (**E**) Male schistosome showing an irregular enlargement of sensory organelles in the tegument (large arrow), formation of quantities of vacuoles (small arrow). (**F**) Female schistosomes with decreased thickness of the dorsal medial tegument showing the failure to close the outer tegument invaginations (arrow indicated). (**G**) Three male worms were randomly selected and the areas occupied by vacuoles were measured by imageJ. (**H**) Three female worms were randomly selected and the thickness of the dorsal medial tegument was analyzed by imageJ.

On TEM, the tegumental structure of parasites incubated with *SjVAMP2* siRNA was distinct from that of the controls. The body wall of *S*. *japonicum* was composed of a tegument, basal lamina, and subtegument. The tegument comprised a syncytial layer of fused cytons connected by cytoplasmic channels. The outer membrane of the tegument was folded and pitted. The tegumental matrix contained many discoid bodies but few membranous bodies. Small vesicles were observed primarily at the infolding membrane of the surface (Fig. 4A–C). Enlarged membranous bodies with heptalaminate membrane were observed in the tegumental matrix of *SjVAMP2*-suppressed male worms (Fig. 4D). And the most significant changes were the presence of extensive vacuoles throughout the surface layer, and enlarged sensory organelles characterized by extensively lysed internal structure, empty internal cavities, or ruptured bilayer membrane (Fig. 4E,G). Despite no external tegumental difference was observed in the *SjVAMP2* siRNA-treated female worms via SEM, the dorsal medial tegument of female worms was consistently thinner than those of the controls (P < 0.01), and the surface invaginations failed to close (Fig. 4F,H).

### Effect of *SjVAMP2* knockdown on glucose uptake

**Figure 5:**
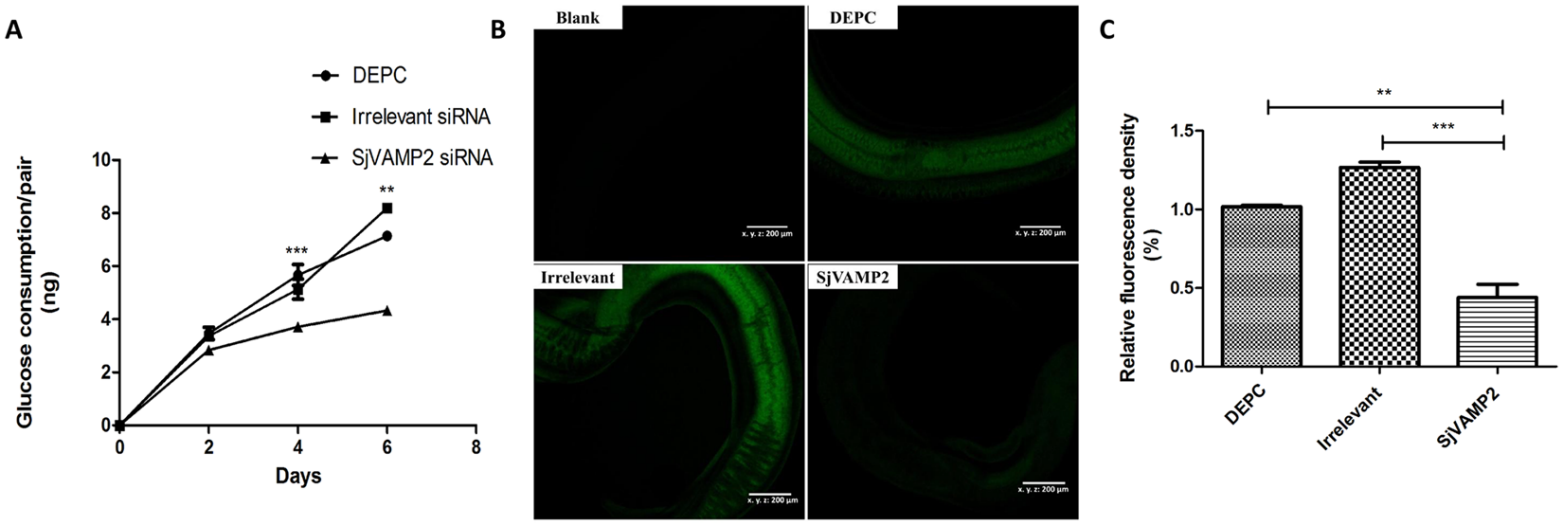
Effect of *SjVAMP2* suppression on glucose uptake. (**A**) Glucose uptake by schistosomes in different groups cultured in low-glucose media for 2, 4 and 6 day, was measured respectively. Data was from three independent experiments, and compared with the DEPC-treated groups (**P < 0.01, ***P < 0.001). (**B**) Confocal laser scanning microscopy observation of *S*. *japonicum* glucose uptake in different groups after siRNA treatment. 2-NBDG was used as a fluorescent probe to determine glucose uptake. Worms not treated with 2-NBDG were used as a negative sample to control for background fluorescence. (**C**) The signals of 2-NBDG were analyzed using imageJ software.

The effect of *SjVAMP2* suppression on glucose uptake was evaluated by measuring the glucose concentration in the worm culture medium in 2, 4 and 6 days, respectively. The glucose consumption of each paired worms in the three groups increased with days going. When the reduction percentage of glucose uptake (compared with the DEPC group) was calculated, there was no significant difference in the three groups in 2 days, but there was a 34.6% (P < 0.001) and 39.3% (P < 0.01) reduction in SjVAMP2 siRNA group in 4 and 6 days, respectively; although it showed a higher glucose uptake in the irrelevant siRNA group in 6 days, there was no significant difference between the two control groups (Fig. 5A). The fluorescent 2-deoxyglucose analog, 2-[N-(7-nitrobenz-2-oxa-1,3-diaxol-4-yl) amino]-2-deoxyglucose (2-NBDG) was used as a sensitive probe to monitor glucose uptake in *SjVAMP2*-suppressed and control worms^46, 47^. Green fluorescence signal was captured via confocal laser scanning microscopy (CLSM) (Supplementary Figure S3) and analyzed. The signal detected in the *SjVAMP2* siRNA-treated worms was not that strong as in the control worms (P < 0.01) (Fig. 5B,C). There was no difference between the irrelevant siRNA-treated and DEPC control group. Moreover, the suppression of *SjVAMP2* gene significantly decreased the expression of four glucose uptake-related genes: *SGTP1* (62.5%, P < 0.01), *SGTP4* (74.7%, P < 0.001), *SjIR1* (51.6%, P < 0.01), and *SjIR2* (66.0%, P < 0.01) (Supplementary Figure S4).

### *SjVAMP2* suppression affected worm development and inhibited egg production

**Figure 6:**
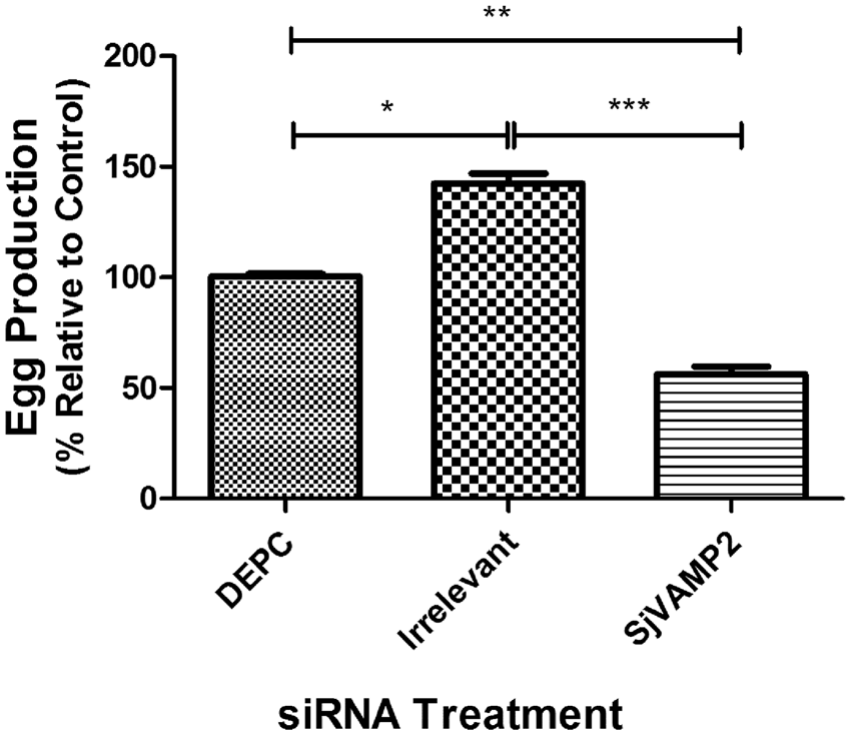
Relative egg production of schistosomes treated with SjVAMP2 specific siRNA *in vitro*. Data were compared with the DEPC-treated groups from three separate experiments. P-values were calculated using t-tests to compare the difference between the RNAi and control groups (*P < 0.05, **P < 0.01, ***P < 0.001).

The effect of *SjVAMP2* suppression on egg production of female schistosomes *in vitro* was evaluated by counting the number of eggs in the 1 mL culture medium with 5 paired worms. Egg production decreased by 43.8% in *SjVAMP2*-suppressed parasites compared with DEPC treated worms (P < 0.01). In the irrelevant siRNA-treated group, egg production increased by 42% compared with DEPC-treated worms (P < 0.05) (Fig. 6). The experiment has been repeated for at least three times, and the results were identical with a high egg production in the irrelevant siRNA control and low egg production in the *SjVAMP2* suppression group.

The effect of the RNAi-mediated suppression of *SjVAMP2* on parasite development *in vivo* was evaluated by treating schistosomes-infected mice with *SjVAMP2* specific siRNA 7 times at every 3 days beginning at 21 dpi, and comparing with that of mice treated with irrelevant siRNA or DEPC under the same condition. The *SjVAMP2* gene expression in *SjVAMP2* siRNA treated group was suppressed by 25.7% (P < 0.05), when compared with the irrelevant siRNA control (Supplementary Figure S5). Table 1 showed the number of worms and eggs per gram (EPG) in livers recovered from mice 42 dpi in two independent experiments. In the first RNAi *in vivo* experiment, there was a 32.8% (P < 0.05) decrease in worm burden and 48.8% (P < 0.05) decrease in hepatic egg burden compared with the irrelevant siRNA-treated group; in the second RNAi *in vivo* experiment, there was a 50.4% (P < 0.01) decrease in worm burden, 40.0% (P < 0.05) decrease in hepatic egg burden compared with DEPC group, and a 38.0% (P < 0.01) decrease in worm burden compared with the irrelevant siRNA-treated group. There was no difference between the irrelevant siRNA-treated and DEPC control group. In addition, liver histopathology of *SjVAMP2* siRNA-treated mice showed remarkable histological improvement compared with the controls (Supplementary Figure S6). Hematoxylin-eosin-stained liver sections from control mice showed characteristic granulomata and hepatic fibrosis, with multifocal lymphohistiocytic infiltrates surrounding schistosome eggs whereas *SjVAMP2*-suppressed mice had fewer and smaller granulomas and improved liver histology with a significant decrease in fibrous tissue.

**Table 1:** Effects of SjVAMP2 gene silencing on worm burden and eggs laying.

Groups (n = 6)	Worm burden (% reduction)	EPG (% reduction)
Experiment 1		
DEPC	20.7 ± 2.9	52974 ± 12427
Irrelevant siRNA	23.3 ± 2.9	67731 ± 9450
SjVAMP2 siRNA	15.7 ± 1.6(24.2%^a^, 32.8%^b*^)	34639 ± 5397(34.6%^a^, 48.8%^b*^)
Experiment 2		
DEPC	19.2 ± 2.5	38234 ± 5664
Irrelevant siRNA	15.3 ± 1.5	31426 ± 4399
SjVAMP2 siRNA	9.5 ± 1.1(50.4%^a**^, 38.0%^b**^)	22931 ± 3789(40.0%^a*^, 27.0%^b^)

Data are presented as the mean ± standard error.

*P < 0.05, ** < 0.01.

^a^Compared with DEPC group.

^b^Compared with Irrelevant siRNA group.

EPG, eggs per gram (EPG) in livers recovered from mice.

## Discussion

Schistosomes can survive for decades within the host bloodstream and evade host immune attack, which is primarily dependent on the properties of the tegument^48, 49^. The tegument surface is a unique double membrane structure, continually rupture and repair^50, 51^. It is known that maintenance of the tegument apical membrane is mediated by the fusion of membranous vesicles with the apical membrane^52^, which is a vesicles trafficking process mediated by SNARE proteins. The molecular mechanisms that support injured axon repair and growth parallel that of synaptic vesicle trafficking and neurotransmitter release within uninjured axons, requiring for SNARE proteins^53, 54^. *Sj*VAMP2 (v- SNARE) has been identified in the tegument of *Schistosoma japonicum* in the previous study^42, 43^, and our study confirmed the location of *Sj*VAMP2 specifically in the tegument apical membrane invaginations and the secretory vesicles in the tegument martrix, which implies the role of *SjVAMP2* in the tegument membrane maintenance or repair process.

When *SjVAMP2* was suppressed in *S*.*japonicum in vitro*, significant morphological changes in the tegumental structure were observed via SEM and TEM. In schistosomes, the ridges and invaginations greatly expanded the tegumental surface and increased the exchange of molecules between the outer environment and internal tissues^18, 55^. We observed that the suppression of *SjVAMP2* resulted in ridges fusion or surface failure in the closure of invaginations, which may inhibit the normal nutrition absorption of the parasites^56^. Besides, tegument peeling on the surface of the anterior portion and enlarged membranous bodies with heptalaminate apical membranes were observed in the tegument of *SjVAMP2*-suppressed male worms. The tegument surface membrane is maintained by membranous vesicles fusing with the apical membrane^57^. Enlarged membranous bodies were observed in the schistosomes after cercariae penetrating the mammalian skin 30 min, transferred from subtegumental cells to the tegument, where they fuse and form the new outer tegument membrane, while shedding off the origin ones of cercariae. During the growth of schistosomula, smaller membranous bodies replace the enlarged membranous bodies and contribute to the continuous renewal of the apical tegument. Study has reported that an increased number of membranous bodies were detected during damage to the outer membrane, presumably to form a new membrane^52^. The enlarged membranous bodies presence in *SjVAMP2*-suppressed worms seem to take the place of the damaged surface and help to form the new tegument surface membranes. This result implies the crucial role *SjVAMP2* played in the maintenance of the tegument during the parasites survival.

Vesicles serve as intracellular transporters between organelles for the release of the stored cargos (hormone, neurotransmitter, nutrients) via membrane fusion^58, 59^. Studies have confirmed the significant role of VAMP2 in exocytosis in non-neuron tissues, including surfactant secretion in the lungs^60^, glucagon-like peptide-1 exocytosis in murine intestinal L cells^25^, and zymogen granule secretion in pancreatic acini^61^. We speculated that suppression of *SjVAMP2* might inhibit the normal cargo trafficking outside or inside the worm tegument, thus increasing the number of sensory organelles on the tegument to compensate the deficiency, and the subsequent malformation of over-sized sensory organelles with vacuolation, degeneration of the underlying syncytium. The observation suggests that *SjVAMP2* may involve in the cargo trafficking on the tegument, and knockdown of *SjVAMP2* affects the initial structure and function of the tegument.

The apical membrane invaginations largely increase the tegumental surface, which is a major site of nutrient uptake^6^. Glucose, amino acids and cholesterol are absorbed from the host blood via the tegument instead of the intestinal epithelium of the parasite, and there are multiple transporter proteins on the tegument^16, 31, 62^. Study has shown that two glucose transporter proteins (SGTP1 and SGTP4) are involved in the glucose transport activity in schistotomes, and SGTP4 localized uniquely to the apical membranes seems to uptake glucose from the host blood, while SGTP1 located on the tegumental basal membrane and the worm body seems to transport the absorbed glucose to the internal tissues^33, 63^. All cells import use glucose transporter proteins (GTPs) to import glucose across their hydrophobic surface membranes^64^. Within insulin stimulation, glucose transporter 4 (GLUT4) in muscle or fat cells translocates from intracellular storage compartments to the cell surface where it transports glucose from the extracellular milieu into the cell, which is a membrane fusion stage^65^. Studies has shown that VAMP2 abundant in all insulin responsive tissues is colocalized with GLUT4, and help the translocation of GLUT4 vesicles via membrane fusion^24, 39^. In our study, we found that suppression of *SjVAMP2* significantly inhibited the glucose uptake ability of worms, as well as the transcript levels of *SGTP1*, *SGTP4*, *SjIR1*, and *SjIR2*. Since the insulin pathway in schistosomes is similar to that of other organisms^34^, and *SGTP1*, *SGTP4*, *SjIR1*, and *SjIR2* have been studied to involve the glucose uptake^35, 36, 66, 67^, we speculated that the low glucose uptake ability induced by *SjVAMP2* knockdown may has a potential connection with suppression of the four genes, even though the precise mechanism involved is unknown. And the location of *Sj*VAMP2 in the worms was similar to that observed for SGTP1 and SGTP4 in *S*. *mansoni* ^63^, which partially support our hypothesis that *SjVAMP2* may be involved in the glucose uptake process of schistosomes.

Our study also showed that the *SjVAMP2*-suppressed worms exhibited a lower viability *in vitro* and lower worm recovery *in vivo*, which may result in the significantly lower egg production of female worms observed. Besides, the decreased glucose uptake of schitosomes in *SjVAMP2*-suppressed group may be also the reason. Since glucose is the major energy source for schistosomes, adult schistosomes has been reported to utilize glucose at a rate equal to 15–25% of their dry weight each hour via mediated transport and diffusion from the tegument^30, 68^. And considerable quantities of glycogen are synthesized continuously, and stored *in vivo*. Once in the absence of a glucose source, schistosomes will rapidly degrade the stored glycogen for survival^69^. Glycogen is also a potential drug target, and niridazole was reported to kill schistosomes by exhausting the glycogen stores in schistosomes^70^. With the importance of glucose, the inability to import glucose by the *SjVAMP2*-suppressed parasites may have a detrimental impact on the viability of worms. Moreover, the reproduction ability of female *S*.*japonicum* is extraordinary strong with an average of 3500 eggs output per day, while it is 300 in *S*. *mansoni* ^71^. Large quantities of molecules are needed to support the egg formation, including amino-acid, glucose, lipid and haem^72, 73^. Study has shown that a deficient diet in the host directly affects egg output and alters the concomitant pathological response^74^. For the development of *S*. *japonicum* eggs *in vitro*, the minimum concentration of glucose required is 0.02 mM^72^. When schistosomes were exposed to glucose-free medium, the uptake of adenine, choline chloride, histidine and lysine were inhibited consequently^75^, which implies the role of glucose in modulation of nutrient influx to the tegument. The low glucose uptake and glucose-induced modulation of nutrient influx, as well as the blocked cargo trafficking induced by *SjVAMP2*-suppression, may hinder the normal molecule transport, and thus resulting in the low egg production of female worms.

In addition, we observed that *SjVAMP2* siRNA treatment had different effects on male and female schistosomes. *SjVAMP2*-suppressed male worms exhibited smaller size, and significant morphological changes, while female worms mainly showed a decreased egg output. Except for the anterior and tail regions, most areas of paired female worms are covered by the males via the gynaecophoral canal, and the tegument primarily consist of flat sheets, occasionally separated by shallow grooves and small pores, which is believed to increase the friction between the male. The surface of male worms is capaciously folded with sponge-like appearance, largely expanding the areas exposed to the outer environment, which can help the increase of nutrition uptake via tegument from the host^6, 18, 55^. At the same time, the exposed surface is generally accepted an ideal target site for the drugs and other adverse factors^17, 76^. In this aspect, the male worms may have more chances to interact with the siRNA treated by soaking, and the original structure of folded membrane invaginations may be damaged by *SjVAMP2* knockdown. Besides, a considerable proportion of the energy required by the female may be indirectly supplied by the male via tegument^77, 78^. Studies have shown that glucose, polypeptides, cholesterol, and glycoproteins are transferred from the male to the female schistosome during mating^79, 80^, and the separated female worms *in vitro* survive not as well as the paired females or the separated male worms under the same condition^81^. And, the glycogen or glucose reserved in the male schistosome can be depleted by the presence of a female in the gynecophoral canal^82^. This is likely means that the males take on additional responsibilities of nutrients uptake for the copulating schistosomes survival, especially in the peak of egg production, and the males would be the first to suffer the detrimental effects of the outer environment. The stunted male parasites induced by the *SjVAMP2* siRNA treatment may be unable to transfer enough nutrients for the females, as well as the inhibition of glucose uptake that contribute to the decrease of egg output.

## Conclusion

Our results indicated that the suppression of *SjVAMP2* gene expression via RNAi affected glucose uptake, as well as the viability and egg output of schistosomes. The morphology of the tegument of *SjVAMP2* siRNA-treated worms was also altered. These findings provide evidence for the key role of *SjVAMP2* in the development of schistosomes.

## Methods

### Ethics statement

All animal experiments were conducted following the guidelines of the Committee for Care and Use of Laboratory Animals of Shanghai Veterinary Research Institute, Chinese Academy of Agricultural Sciences (Permit Number: SHVRI-2013-0909). The protocol was approved by the Ethics and Animal Welfare Committee of the Shanghai Veterinary Research Institute, Chinese Academy of Agricultural Sciences.

### Parasites and animals

Snails (*Oncomelania hupensis*) naturally infected with *S*. *japonicum* (Anhui isolate) were reared in our laboratory. Pathogen-free male BALB/c mice aged 6 weeks, purchased from the Shanghai Laboratory Animal Center, Chinese Academy of Agricultural Sciences (Shanghai, China), were percutaneously infected with 20 to 40 cercariae of *S*. *japonicum*. The schistosomes were collected from BALB/c mice at 21dpi.

### Localization of SjVAMP2 on the tegument

Sections of the tegument of 21-day-old schistosomes were blocked with 10% bovine serum albumin at 4 °C overnight, and then probed with specific mouse anti-*Sj*VAMP2 specific serum^43^ for 2 h at room temperature. Following six 5-min washes with phosphate-buffered saline (PBS, pH 7.4), the tissue sections were incubated with 10-nm colloidal gold-conjugated goat anti-mouse IgG (Abcom, Cambridge, UK) (1:40 dilution) for 1 h. These sections were washed 5 times with PBS and then twice in ddH_2_O, stained with 5% uranyl acetate (System Biosciences, Mountain View, CA, USA) for 5 min, washed twice in ddH_2_O, and stained with lead citrate (System Biosciences) for 1 min. After that, the sections were washed in ddH_2_O, dried, and visualized by TEM (Hitachi H-7600, Tokyo, Japan).

### Parasite cultures and siRNA treatment *in vitro*

The RNAi technique^83, 84^, which is feasible for schistosomes, was used to characterize the function of *SjVAMP2* (GenBank Accession No. AAP05935.1). The parasites were obtained by portal perfusion using preheated RPMI 1640 medium (HyClone, Logan, UT, USA) from mice at 21 dpi, and incubated in a six-well flat bottom plate (approximately 15 worm pairs/well) containing 2 mL of RPMI-1640 medium (2 g/L glucose) supplemented with 10% (v/v) heat-inactivated fetal calf serum (Gibco, GrandIsland, New York, USA), 100 IU/mL of penicillin, and 100 mg/mL of streptomycin (Sigma, St. Louis, MO, USA), at 37 °C in an atmosphere of 5% CO_2_. Three siRNAs (S1, S2, S3) that targeted different regions of the *SjVAMP2* gene were designed and chemically synthesized in Shanghai GenePharma, Shanghai, China (Supplementary Table S1). An irrelevant siRNA was synthesized as a negative control, and its sequence did not match any sequence deposited in the *S*. *japonicum* genome. *SjVAMP2*-specific or irrelevant siRNA (final concentration of 200 nM)^85^ was delivered to the cultured parasites by soaking in the 2 mL culture media. DEPC water was given under the same condition as a control group. Worms were given fresh medium with siRNA every two days and harvested at 6 days for analysis. The viability of schistosomes was measured by adding 0.1 mg/mL Hoechst 33258 (Yisheng, Shanghai, China) to the cultures, and dead parasites were counted using a confocal laser scanning microscopy (CLSM, Nikon, Tokyo, Japan). And the morphology of schistosomes was captured via an inverted microscope (Olympus, Japan). RNAi experiments were repeated independently three times. Gene suppression was assessed by comparing the mRNA and protein expression levels in the target and control groups.

### Real time PCR analysis of SjVAMP2 gene expression

The transcriptional levels of *SjVAMP2* in siRNA-treated worms were measured by real-time PCR. At day 6 after treatment, total RNA was extracted from worms by using trizol (Invitrogen, Carlsbad, CA, USA) and then transcribed into cDNA using the PrimeScript RT reagent Kit (TaKaRa, Osaka, Japan) according to the manufacturer’s instructions. PCR amplification was performed using the SYBR Premix Ex TaqTM kit (TaKaRa) on an ABI PRISM 7500 Fast Real-Time PCR System (Life Technologies). Nicotinamide Adenine dinucleotide Hydrogen (NADH), which has been showed to have a constant transcriptional level, was used as an endogenous control^86^. The primer sequences are presented in Supplementary Table 2, and the specificity of each sequence was confirmed by BLAST analysis. The experiments were performed independently three times and the 2^−ΔCt^ method was used to calculate the relative expression^87^. One *SjVAMP2*-specific siRNA with high silencing efficiency was selected for use in the subsequent experiments.

### Western blotting analysis

At 6 days post-siRNA treatment, soluble proteins were extracted from worms and quantified by using the Bradford protein assay (Sangon Biotech, Shanghai, China). Subsequently, the proteins were separated by SDS-PAGE, electrotransferred onto a PVDF membrane (Whatman International Ltd., Kent, UK), and blocked in 5% skim milk in PBS (pH 7.4) containing 0.1% Tween 20 (Sigma, St Louis, MO, USA) (PBST) overnight at 4 °C. The membrane was incubated with specific mouse anti-r*Sj*VAMP2 serum^43^ at a dilution of 1:100 or β-actin (Cell Signaling Technology, Boston, MA, USA) diluted 1:1000 in PBST for 1 h at room temperature. After three 5-min washes in PBST, the bound primary antibodies were detected using horseradish peroxidase-conjugated goat anti-mouse IgG (Beyotime Biotechnology, Shanghai, China) diluted 1:2000 for 1 h at room temperature. The membrane-bound proteins were exposed to an X-ray film and detected using the enhanced chemiluminescence detection system (GE Healthcare Life Sciences, Stockholm, Sweden).

### Effect of *SjVAMP2* suppression on the tegument of schistosomes

Both SEM and TEM were employed to observe the tegument of siRNA-treated and control schistosomes. At 6 days post treatment, worms were collected and washed in PBS (pH 7.4) three times, and immediately chemically fixed with 4% paraformaldehyde and 2.5% glutaraldehyde (System Biosciences, Mountain View, CA, USA) at 4 °C for 48 h, followed by osmium tetroxide (System Biosciences, Mountain View, CA, USA) at room temperature. Then the worms were dehydrated in increasing concentrations (from 30% to 100%) of acetone (TiTan, Shanghai, China). For SEM, the samples were freeze-dried and coated with platinum (System Biosciences, Mountain View, CA, USA) by sputtering with a plasma multicoater (PMC-5000; Meiwafosis, Tokyo, Japan). For TEM, the dehydrated samples were embedded into resins (TiTan, Shanghai, China), and then thin sections were cut, transferred to metal grids (Agar Scientific, Essex, UK). Uranyl acetate (System Biosciences, Mountain View, CA, USA) was used for postsection staining. Images were captured with a scanning electron microscope (JSM-6390LV, JEOL, Tokyo, Japan) in high-vacuum mode with an accelerating voltage of 2–10 kV, or with a transmission electron microscope (Hitachi H-7600, Tokyo, Japan) with an accelerating voltage of 80 kV, respectively.

### Analysis of glucose uptake and parasite fecundity

Six days after treatment with specific siRNA, five pairs of worms were thoroughly and gently washed three times in PBS and transferred to a new well containing 1 mL of fresh low-glucose (1 g/L) DMEM (Gibco). At day 2, 4 and 6, 100 μl of culture medium was collected and the glucose concentration was measured using a glucose assay kit (Biocore, Gaithersburg, MD, USA) with the detection sensitivity in the range 1–10,000 μM, respectively. Low-glucose DMEM without worms was prepared under the same conditions as the blank control. Glucose uptake of *SjVAMP2*-suppressed worms was calculated from triplicate experiments relative to DEPC control group. At the same time, the worms were collected and washed three times in PBS and transferred to a new well containing 1 mL of fresh DMEM medium (without glucose) supplemented with 200 μM 2-NBDG, which served as a fluorescence probe to detect glucose uptake in parasites^47^. After incubation for 30 min, the worms were washed three times in PBS and fixed with 4% paraformaldehyde for 30 min, and the fluorescence signal was recorded by CLSM (Nikon). Concomitantly, the number of eggs in the culture low glucose DMEM and wash media was counted using an inverted microscope (Olympus).

### Expression analysis of glucose uptake-related genes in *S*. *japonicum*

As described above, real time PCR was also performed to determine the transcriptional levels of four glucose uptake-related genes: *SGTP1*, *SGTP4*, *SjIR1*, and *SjIR2* ^33, 34, 67^ in worms after treatment with siRNA. *NADH* was used as a reference gene^86^. The accession numbers and primer sequences of these genes are provided in the supporting information (Supplementary Table 2). The results are shown as the gene knocked-down expression levels relative to the DEPC control group.

### Effect of *SjVAMP2* suppression on schistosomes from infected mice

Four groups of 6-week-old male BALB/c mice, with 6 mice per group, were infected with 35 ± 2 freshly shed cercariae per mouse. At 21 dpi, each mouse in each group received a 0.1 mL injection of DEPC water, 33 μg irrelevant siRNA (dissolved in 0.1 mL DEPC), or 33 μg *SjVAMP2* siRNA (dissolved in 0.1 mL DEPC) via the caudal vein and this was repeated every 3 days, 7 injections in total. At 42 dpi, the mice were sacrificed, and the number of worms recovered from each mouse (worm burden) was recorded. Concomitantly, the liver of each mouse was removed and divided evenly into two parts: one part was used to calculate the eggs and the other part was washed in PBS (pH 7.4), fixed in 4% paraformaldehyde, processed, and stained with hematoxylin and eosin. Specific mouse anti-r*Sj*VAMP2 serum^43^ was used for immunohistochemistry staining^88^. The hematoxylin-eosin-stained sections were examined under an optical microscope (Olympus). The worms recovered were used for *SjVAMP2* gene expression analysis as well. A repeat trial was performed under the same condition 3 months later.

### Statistical analysis

All data acquired in this study were generated from at least three replicates of independent experiments using identical protocols, and are presented as the mean ± SE. GraphPad Prism 5 software (GraphPad software Inc, CA) was used for statistical analysis. The t-test was used to compare the means between the target and control groups, and P-values smaller than 0.05 and 0.001 were considered significant and highly significant, respectively.

## Electronic supplementary material

Supplementary information
