# Supplementary material for: Suppression of VAMP2 Alters Morphology of the Tegument and Affects Glucose uptake, Development and Reproduction of Schistosoma japonicum
Source: Sci Rep. 2017 Jul 12;7:5212. doi: 10.1038/s41598-017-05602-8 (PMC5507895; doi:10.1038/s41598-017-05602-8)

Suppression of VAMP2 Alters Morphology of the Tegument and Affects Glucose uptake, Development and Reproduction of *Schistosoma japonicum*

Qian Han[1], Bingguang Jia[1], Yang Hong[1], Xiaodan Cao[1], Qi Zhai[1], Ke Lu[1], Hao Li[1], Chuangang Zhu[1], Zhiqiang Fu[1], Yonghong Shi[1], Jiaojiao Lin[*1,2]

[1]Ministry of Agriculture, Shanghai Veterinary Research Institute, Chinese Academy of Agricultural Sciences, Shanghai, People's Republic of China

[2]Jiangsu Co-innovation Center for Prevention and Control of Important Animal Infectious Diseases and Zoonoses, Yangzhou, China

* The Corresponding Author: Jiaojiao Lin

Tel: +86 21 3429 3440

Fax: +86 21 5408 1818

E-mail: jjlin@shvri.ac.cn

Supplementary Table S1. Sequences of SiRNAs.

Name	Sequence	Targeting regions of SjVAMP2
S1 siRNA	Sense 5'-GCGGCUACAAACUCAATT-3'<br>Antisense 5'-UUGAGUUUGUAGCCGCTT-3'	69-87
S2 siRNA	Sense 5'-GCUUCACAAUUCGAGGCUATT-3'<br>Antisense 5'-UAGCCUCGAAUUGUGAAGCTT-3'	199-217
S3 siRNA	Sense 5'-GCAUUCUGCACCAAGUCAUTT-3'<br>Antisense 5'-AUGACUUGGUGCAGAAUGCTT-3'	381-399
Irrelevant siRNA	Sense 5'-UUCUCCGAACGUGUCACGUTT-3'<br>Antisense 5'-ACGUGACACGUUCGGAGAATT-3'	no

Supplementary Table S2. Primers of genes for real-time PCR.

Gene	Abbreviation	ID of gene	primers
vesicle-associated membrane protein 2	VAMP2	AAP05935.1	Forward: 5'-ACAACCTCGACCACAGAACAAG-3'<br>Reverse: 5'-TTCCTGCACTAGCCTCGAATTG-3'
Insulin receptor 1	IR1	GQ214553	Forward: 5'-TTCAGTTAATTGACGAGAATATTGA-3'<br>Reverse: 5'-TCCTATTCTAGTATGATTGGACTCTGA-3'
Insulin receptor 2	IR1	GQ214554	Forward: 5'-TCAGTATCATCCTCATCACCAAA-3'<br>Reverse: 5'-ATGCATCATCAACAGGCGTA-3'
Glucose transporter protein 1	GTP1	Sjp_0093040.1	Forward: 5'-GCAGGTGCAATAGGAGCATT-3'<br>Reverse: 5'-CCAATAATAAAGCGGCCAAT-3'
Glucose transporter protein 4	GTP4	Sjp_0132820.1	Forward:5'-TAAGCTCTTTACTCAGAAAGATTTACGTATGC-3'<br>Reverse:5'-AACAACACAAAACTGTATGTAGTCAAGTGGGAT-3'
Nicotinamide Adenine dinucleotide Hydrogen	NADH	Q9B8Z5-1	Forward: 5'-CGAGGACCTAACAGCAGAGG-3'<br>Reverse: 5'-TCCGAACGAACTTTGAATCC-3'

Supplementary Figure S1. Diagram of the adult schistosome tegument and associated cell bodies.

(http://www.sciencedirect.com/science/article/pii/S0065308X06630030)

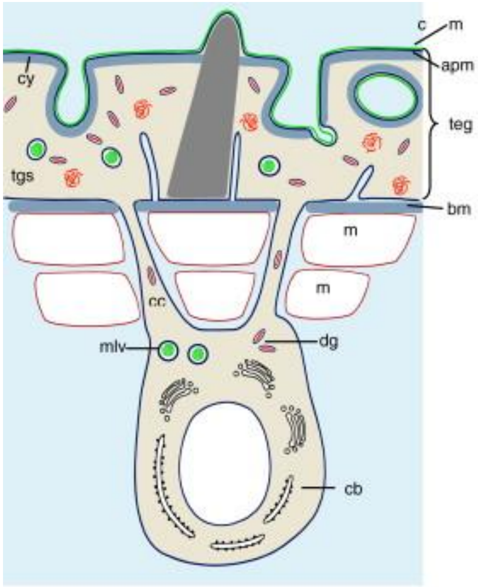

apm	apical plasma membrane
bm	basement membrane
cb	cell body
cc	cytoplasmic connection
cy	cytoskeleton
dg	discoid granules
m	muscle fibres
mc	membranocalyx
mlv	multilaminate vesicles
teg	tegument
tgs	tegument ground substance

Supplementary Figure S2. Immunolocalization of SjVAMP2 on the tegument of schistosomes. **(A).** Gold particles immunoreactive for SjVAMP2 were observed on the tegument (particularly in its invaginations, i), basal membrane (BL) and the underlying muscles (M). **(B).** In the tegumental matrix, gold particles were mainly distributed at apical membrane invaginations (i), discoid bodies (Db), and membranous bodies (Mb).

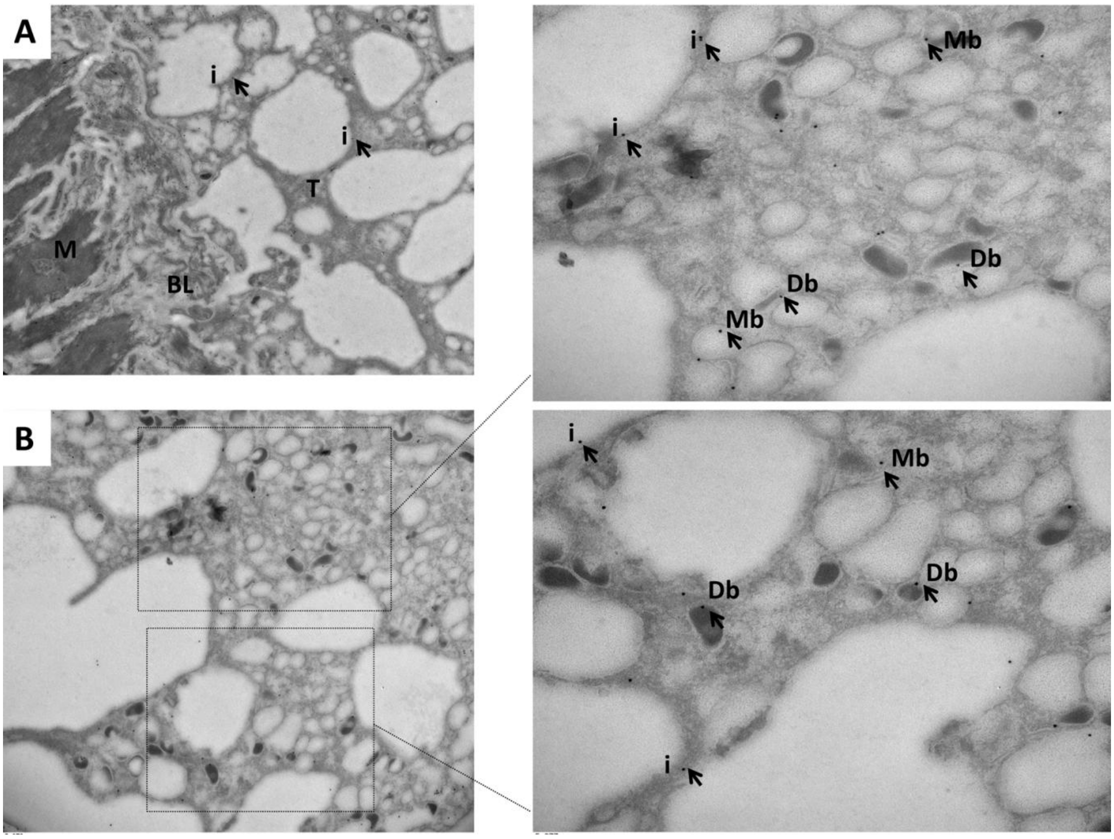

Supplementary Figure S3. Z-stack of *S. japonicum* fluorescence signals after treatment with DEPC, irrelevant siRNA, or SjVAMP2 siRNA.

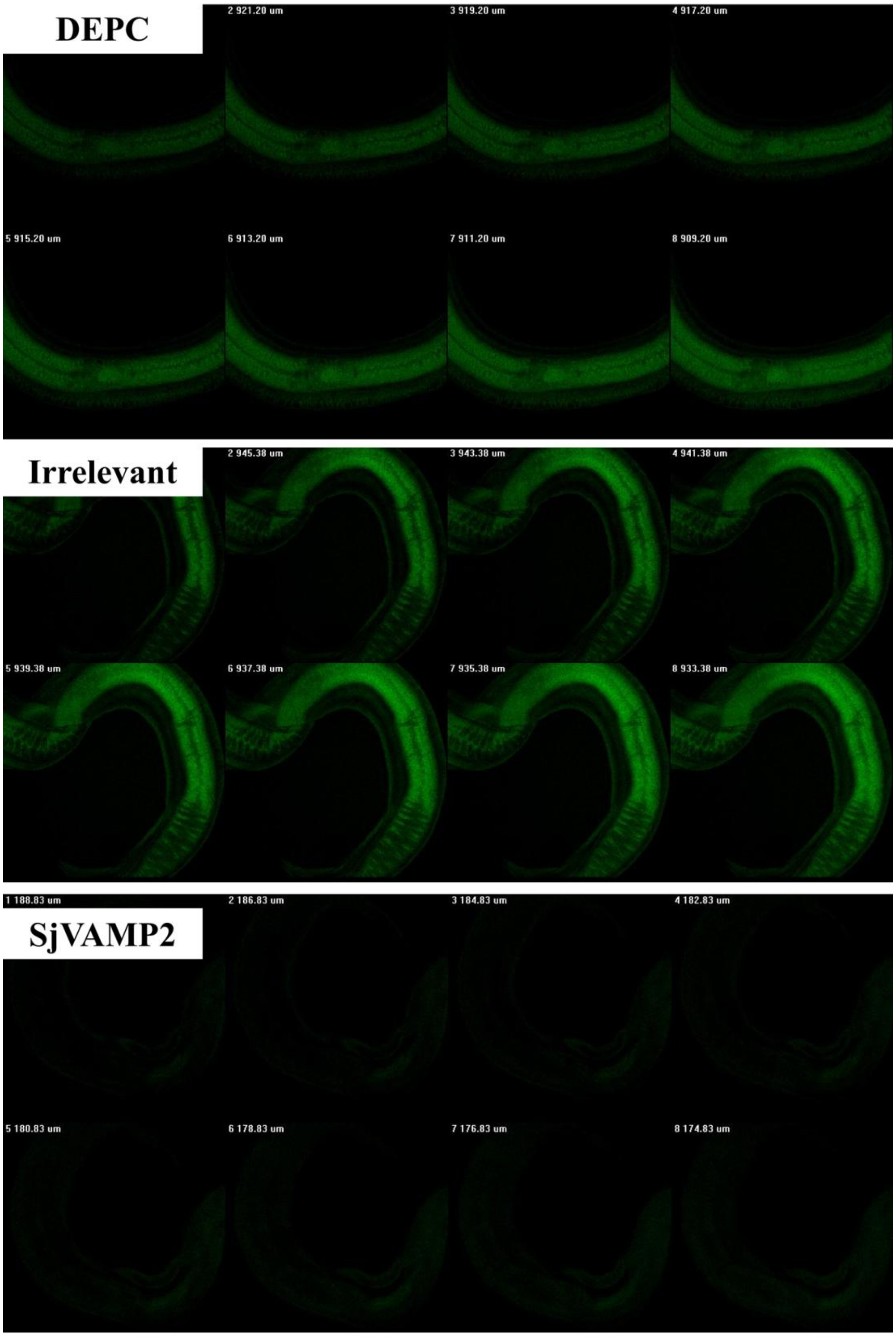

Supplementary Figure S4. Modulation of transcript levels of four genes involved in glucose uptake in *S. japonicum* after treatment with SjVAMP2 siRNA.

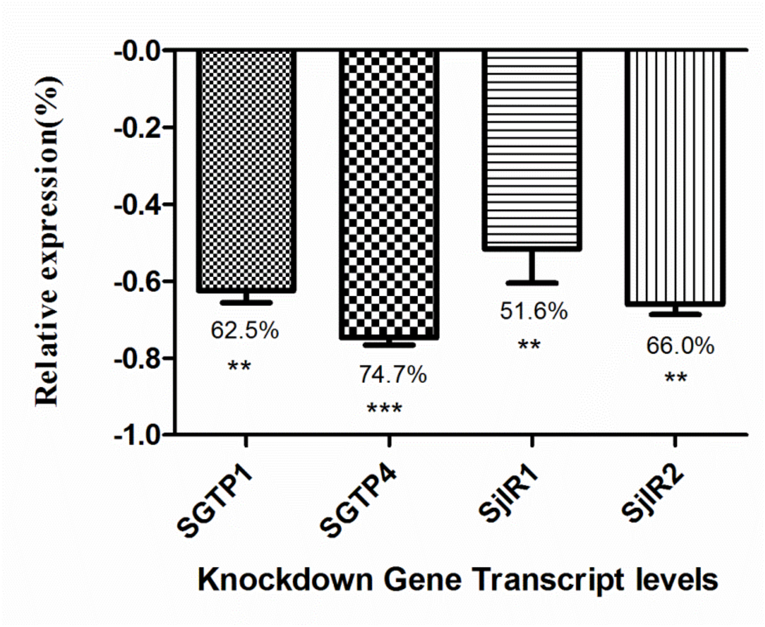

Supplementary Figure S5. Expression of SjVAMP2 in *S. japonicum* after treatment with indicated siRNA *in vivo*.

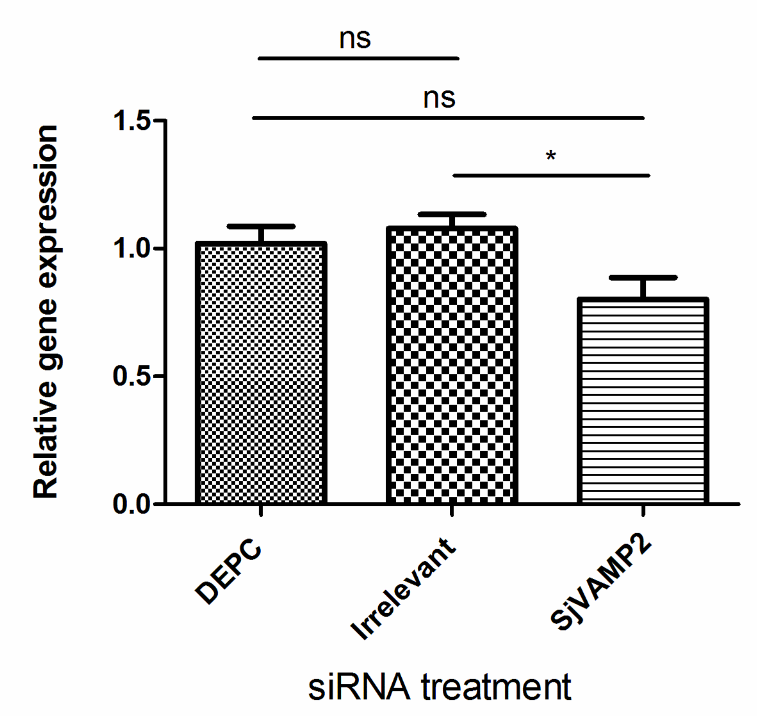

Supplementary Figure S6. Histopathology of livers from mice in different groups and the size of granulomas was analyzed by image J. Livers of mice not infected with *S. japonicum* were used as a negative control whereas the other groups infected of *S. japonicum* were treated with irrelevant siRNA (control), or SjVAMP2 siRNA, respectively.

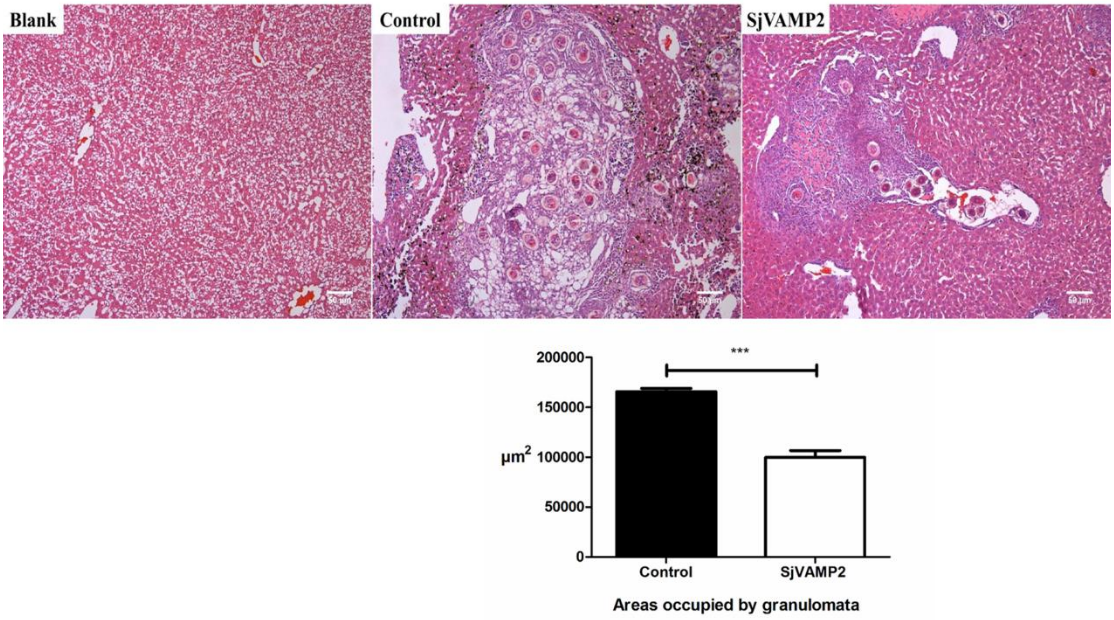

Supplement: Supplementary file 1 — Supplementary information [file 41598_2017_5602_MOESM1_ESM.pdf]
